# Short-Term Protocols to Obtain Insulin-Producing Cells from Rat Adipose Tissue: Signaling Pathways and In Vivo Effect

**DOI:** 10.3390/ijms20102458

**Published:** 2019-05-18

**Authors:** Krista Minéia Wartchow, Letícia Rodrigues, Lucas Zingano Suardi, Barbara Carolina Federhen, Nicholas Guerini Selistre, Carlos-Alberto Gonçalves, Patrícia Sesterheim

**Affiliations:** 1 Federal University of Rio Grande do Sul (UFRGS), Biochemistry Post-Graduate Program, Porto Alegre 90035003, Brazil; kristawartchow@gmail.com (K.M.W.); letigues@gmail.com (L.R.); lucas_zingano@hotmail.com (L.Z.S.); barbarafederhen@gmail.com (B.C.F.); nicholas.gueriniselistre@gmail.com (N.G.S.); 2 Institute of Cardiology of Rio Grande do Sul, Experimental Center, Porto Alegre 90650-090, Brazil; patriciasester@gmail.com

**Keywords:** adipose-derived stromal cells, exendin-4, diabetic rats, insulin-producing cells, p38-MAPK, PI3K/Akt

## Abstract

Studies using mesenchymal stromal cells (MSCs) as a source of insulin-secreting cells (IPCs) are a promising path in the pursuit for diabetes therapy. Here, we investigate three short-term differentiation protocols in order to generate IPCs from autologous adipose-derived stromal cells (ADSCs) with an expressive insulin-secreting profile in vitro and in vivo, as well as the signaling pathways involved in the chosen differentiation protocols. We extracted and cultured ADSCs and differentiated them into IPCs, using three different protocols with different inductors. Afterwards, the secretory profile was analyzed and IPCs differentiated in exendin-4/activin A medium, which presented the best secretory profile, was implanted in the kidney subcapsular region of diabetic rats. All protocols induced the differentiation, but media supplemented with exendin-4/activin A or resveratrol induced the expression and secretion of insulin more efficiently, and only the exendin-4/activin-A-supplemented medium generated an insulin secretion profile more like β-cells, in response to glucose. The PI3K/Akt pathway seems to play a negative role in IPC differentiation; however, the differentiation of ADSCs with exendin-4/activin A positively modulated the p38/MAPK pathway. Resveratrol medium activated the Jak/STAT3 pathway and generated IPCs apparently less sensitive to insulin and insulin-like receptors. Finally, the implant of IPCs with the best secretory behavior caused a decrease in hyperglycemia after one-week implantation in diabetic rats. Our data provide further information regarding the generation of IPCs from ADSCs and strengthen evidence to support the use of MSCs in regenerative medicine, specially the use of exendin-4/activin A to produce rapid and effectively IPCs with significant in vivo effects.

## 1. Introduction

Type 1 diabetes mellitus (DM1), characterized as a multifactorial disease dependent on the complex interaction between predisposing genetic factors, immune response, and environmental influences, is a chronic autoimmune disease resulting in the destruction of pancreatic β-cells [1] and, consequently, in the loss of insulin production and secretion [2]. There has been increasing interest and progress in the regenerative therapies field, including studies focusing on the generation of insulin-producing cells (IPCs) derived from embryonic stem cells, the umbilical cord, and various adult tissues, such as adipose tissue [3,4], since insulin administration does not prevent the long-term complications of diabetes. 

The use of mesenchymal stromal cells (MSCs) as a source of β-cells has been pursued and many successful in vitro attempts have been made to differentiate MSCs in IPCs. Chen et al. [5] reported the first in vitro differentiation of MSCs into cells that were similar to pancreatic islets and which, when transplanted, proved to be functional in the control of blood glucose concentrations in diabetic rats [6]. Since then, numerous modified protocols employing different stimulatory agents have been tested in order to increase the effectiveness of IPC differentiation [7,8]. Chandra et al. [9] reported the generation of IPCs derived from adipose tissue (adipose-derived stromal cells (ADSCs)), which have been shown to be an ideal population of stem cells for cell therapy since they are found in abundance, are of the easiest availability and represent an autologous tissue. Transplantation of autologous MSCs would help overcome the major limitations of inadequate delivery and/or allogeneic rejections since they have an immunomodulatory effect in suppressing the immune response in autoimmune and inflammatory diseases [10,11]. 

Differentiation of IPCs from MSCs can be carried out using many inductors. Nicotinamide has been used to generate IPCs from different cell sources (e.g., [11,12]). More recent studies have shown that exendin-4 (EX-4) acts on the differentiation of MSCs in IPCs [13,14,15]. Interestingly, EX-4 is a long-acting GLP-1 receptor agonist (glucagon-like peptide 1) that acts directly on pancreatic β cells, increasing insulin secretion [16], with long-term beneficial effects on blood glucose levels [17] and on cognitive deficit in diabetic rats [18]. Another potential inductor is resveratrol (RSV), a naturally occurring polyphenolic compound found in various fruits and plants [19]. Studies have shown that RSV facilitates cardiomyocyte and osteogenic differentiation of embryonic stem cells and induced pluripotent stem cells and is able to prevent cell apoptosis by decreasing levels of reactive oxygen species [20,21]. RSV can also increase the proliferation and differentiation of human mesenchymal stromal cells [22]. In addition to its ability to promote osteogenic differentiation, RSV is also capable of stimulating the differentiation of embryonic stem cells into cardiomyocytes [21,23]. 

The signaling pathways involved in the cell differentiation of IPCs are not fully understood. The Jak/STAT pathway, commonly activated by cytokine signaling, is known to play an important role in β-cell differentiation [24,25,26]. p38 Kinase, a member of MAPK family, has also been implicated in the differentiation of β-cells [27,28]. In addition, PI3K/Akt activation plays a critical role in the differentiation of pancreatic duct cells into IPCs during pancreatic regeneration [29] and in the survival of these cells [30]. All these three signaling pathways participate in the differentiation of IPCs, but their participation in the route stimulated by the protocols of IPC generation mentioned above have not been evaluated, in particular for the differentiation of IPCs from ADSCs.

Therefore, the aim of this work was to produce IPCs from ADSCs using short-term differentiation protocols with an effective insulin secretion profile as well as identify the signaling pathways involved in this initial phase of induction. Besides the in vitro insulin secretion, we investigated the possible in vivo effect after an implant of the IPCs based on profile of insulin secretion and glycemia control in diabetic rats.

## 2. Results

### 2.1. Morphological and Phenotypic Characterization of ADSC Culture 

**Figure 1:**
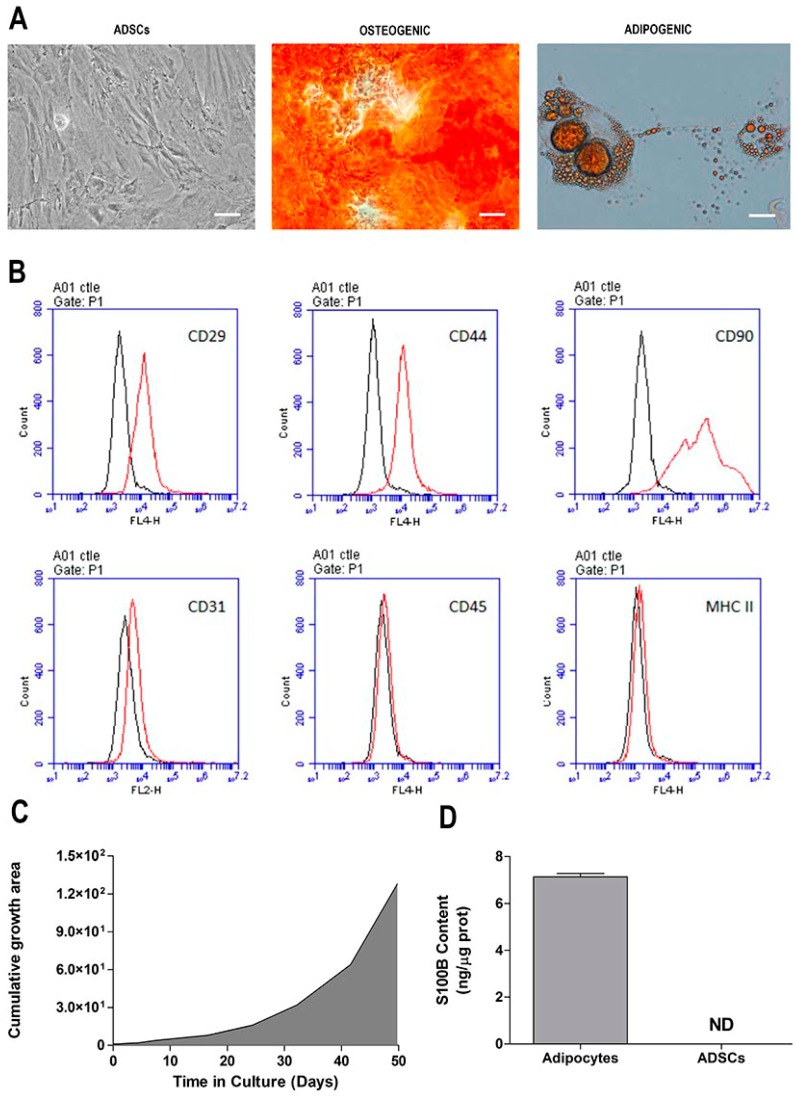
Morphological and phenotypic characterization of adipose-derived stromal cell (ADSC) cultures. (**A**) Cell morphology (first panel) and adipogenic and osteogenic differentiation of ADSCs (second and third panels, respectively) at 40× magnification using phase-contrast microscopy. Scale bar = 30 µM; (**B**) immunophenotyping of ADSCs by flow cytometry; peaks are the expression of the selected molecules (black trace), compared to the negative isotype control (red trace); (**C**) curve of ADSC in vitro growth over time; (**D**) S100B protein content in ADSCs and adipose tissue, as measured by ELISA. Representative data of 6 independent experiments performed in triplicate.

After reaching 80% confluence, stromal cells derived from adipose tissue were trypsinized and passaged every 4 to 5 days. No morphological changes were observed during the p1–p5 passages. ADSCs presented a fibroblast-like shape, and a homogeneous and monolayered growth (Figure 1A, first panel). Analysis of the in vitro expansion of the mesenchymal stromal cells showed a proportional growth curve over time (Figure 1C). For immunophenotyping, surface antigens from ADSCs were analyzed by flow cytometry (Figure 1B). The immunophenotypic analysis showed that ADSCs expressed molecular markers such as CD29, CD44, and CD90 (Thy-1) on their surface. Moreover, the cells did express typical endothelial cell markers, such as CD31, CD45, and MHC II. We also evaluated the adipogenic and osteogenic differentiation of the ADSCs. After the cells were cultured in adipogenic medium for four weeks; Red Oil-positive lipid droplets without cytoplasm were formed (Figure 1A, second panel). When ADSCs were maintained in osteogenic medium for 4 weeks, they presented calcium deposits that could be stained with Alizarina Red S, a dye that stains the calcium-rich extracellular matrix (Figure 1A, third panel). As the mesenchymal stromal cells were isolated from the epididymal adipose tissue of Kyoto rats, the S100B protein content was evaluated in the ADSCs (Figure 1D) (*p* < 0.0001; *t* = 47.53; *df* = 10). This protein is highly expressed (and secreted) by adipose tissue, but is clearly not expressed in ADSCs. 

### 2.2. Differentiation of ADSC into Insulin-Producing Cells (IPCs)

**Figure 2:**
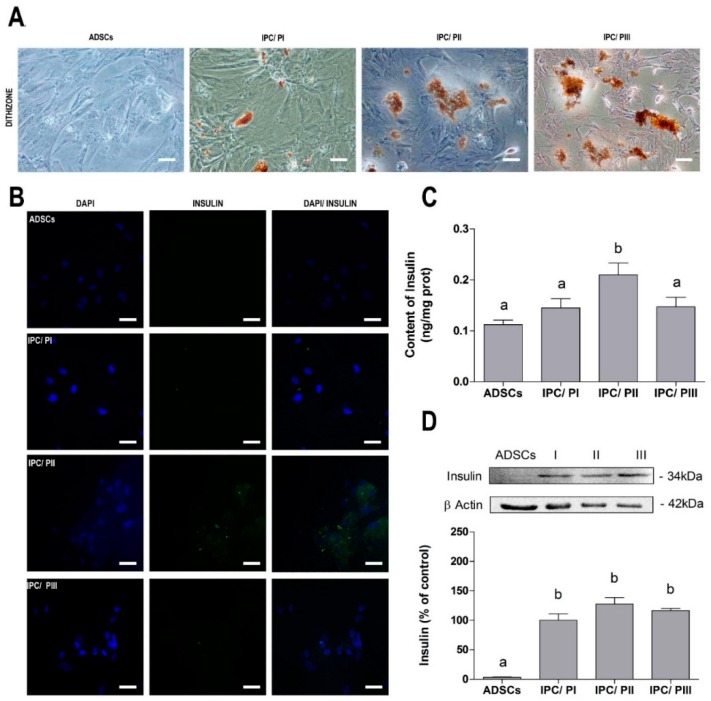
Differentiation of ADSCs into insulin-producing cells (IPCs). (**A**) Dithizone (DTZ) staining for indirect insulin assessment at 20× magnification by phase-contrast microscopy. Scale bar = 60 µM; (**B**) immunofluorescence for insulin (green) and DAPI nuclear staining (blue), at 40× magnification by confocal microscopy. Scale bar = 30 µM; (**C**) insulin content, measured by ELISA; and (**D**) pro-insulin content, evaluated by Western blotting. Data are expressed as means ± SE of 6 independent experiments performed in triplicate. Letters indicate different statistical groups by ANOVA followed by Tukey’s test, assuming *p* < 0.05.

ADSCs were submitted to three different differentiation protocols to obtain IPCs. After the differentiation period, the IPCs were stained with DTZ, an indirect marker of insulin. Undifferentiated cells were observed to be DTZ dye negative, whereas the ADSCs submitted to the differentiation protocols were positive for DTZ, particularly following protocols II (supplemented with Exendin-4 and Activin A) and III (supplemented with resveratrol) (Figure 2A). Immunofluorescent images for anti-insulin showed that cells exposed to PII presented a higher fluorescence intensity, whereas the fluorescence was less intense in cells that underwent PI or PIII (Figure 2B). The higher expression of insulin in the IPCs submitted to PII was confirmed by ELISA (Figure 2C) (*p* < 0.0001; f_(7.54)_ = 6.240). Proinsulin content was evaluated by Western blotting (Figure 2D) (*p* = 0.0020; f_(3.20)_ = 7.061). 

### 2.3. Transport of Glucose in IPCs

**Figure 3:**
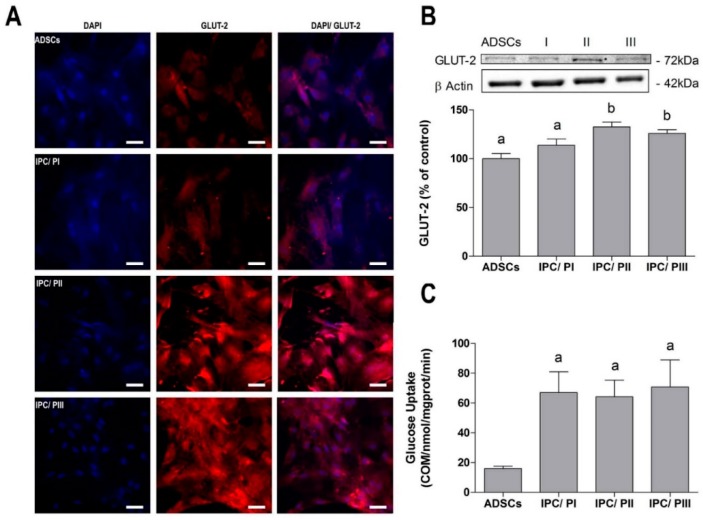
Evaluation of glucose transport in IPCs. (**A**) Immunofluorescence for GLUT-2 glucose transporter (red) and DAPI nuclear staining (blue), at 40× magnification by confocal microscopy. Scale bar = 30 µM; (**B**) evaluation of GLUT-2 expression by Western blotting; and (**C**) evaluation of glucose uptake of 0.1 μCi/mL [2,3-^3^H] deoxy-d-glucose. Data are shown as means ± SE of 6 independent experiments performed in triplicate. Letters indicate different statistical groups by ANOVA followed by Tukey’s test, assuming *p* < 0.05.

Immunofluorescence detection of the glucose transporter GLUT-2 was slightly higher in the cells that underwent PII or PIII (Figure 3A), and this was confirmed by Western blotting (Figure 3B) (*p* = 0.0015; f_(3.20)_ = 7.461). However, glucose uptake by IPCs was equally and significantly increased in cells that underwent all three differentiation protocols, in comparison to the non-differentiated cells (Figure 3C) (*p* = 0.0027; f_(3.15)_ = 7.484).

### 2.4. Secretion of Insulin in Response to Glucose Stimulation in IPCs

**Figure 4:**
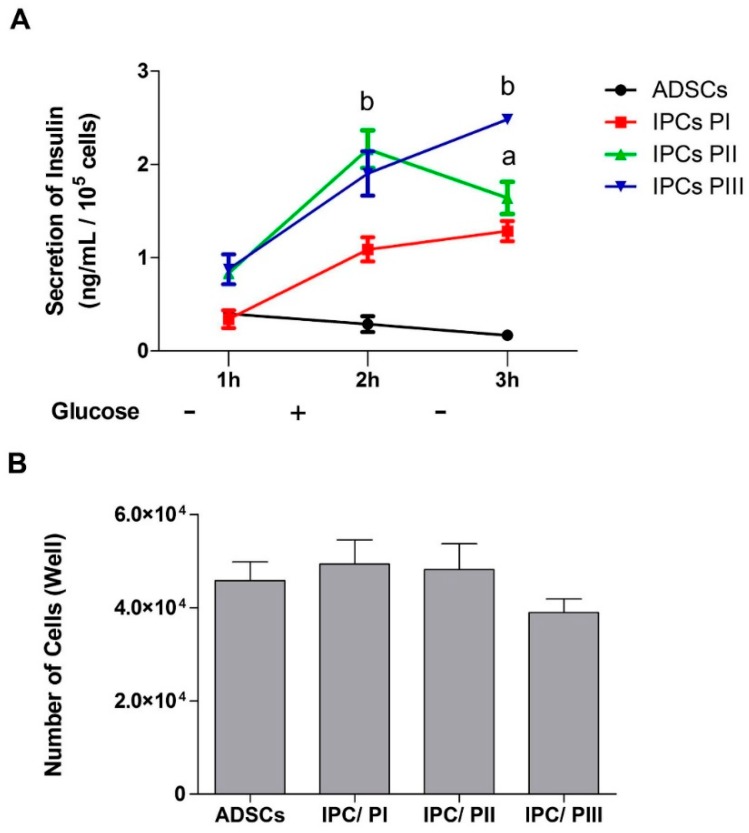
Insulin secretion by IPCs in response to glucose stimulation. (**A**) Insulin secretion curve in response to absence (−) or presence (+) of glucose (at 17.5 mM), as measured by ELISA. (**B**) Quantification of cells by the trypan blue exclusion method. The degree of cell integrity was determined by staining cells with Trypan blue. Stained cells were observed between 30 s and 2 min after the addition of Trypan blue using a Nikon Phase-contrast inverted light microscope at 100× magnification. In each sample, two fields of 100 cells were counted and the mean percentage of stained permeabilized cells was calculated. Data are expressed as means ± SE of 6 independent experiments performed in triplicate. Letters indicate different statistical groups in relation to undifferentiated ADSCs by repeated measures ANOVA (**A**) or one-way ANOVA (**B**) followed by Tukey’s test, assuming *p* < 0.05.

The supernatants of cell cultures (ADSCs and IPCs) were collected at 1h after three consecutive media changes (DMEM without glucose—glucose 0; DMEM F12—glucose at 17.5 mM; and again, DMEM without glucose) and secreted insulin was evaluated by ELISA (Figure 4A). Results indicate that the undifferentiated ADSCs did not contain/release insulin and did not respond to the glucose stimulus. The IPCs differentiated by PI clearly responded to the stimulus, but the insulin secretion was higher for IPCs submitted to PII or PII (*p* = 0.0239; f_(3.11)_ = 6.732), which responded by significantly increasing glucose concentration into the medium. However, when the stimulus was withdrawn, only the IPCs submitted to PII showed a decrease in insulin secretion. The cell number was quantified by the Trypan blue exclusion method to be sure that observed differences were not due to the different number of surviving cells after media changes (Figure 4B) *(p* = 0.4310; f_(3.18)_ = 0.9644).

### 2.5. IPC Signaling Involved in Insulin Expression 

**Figure 5:**
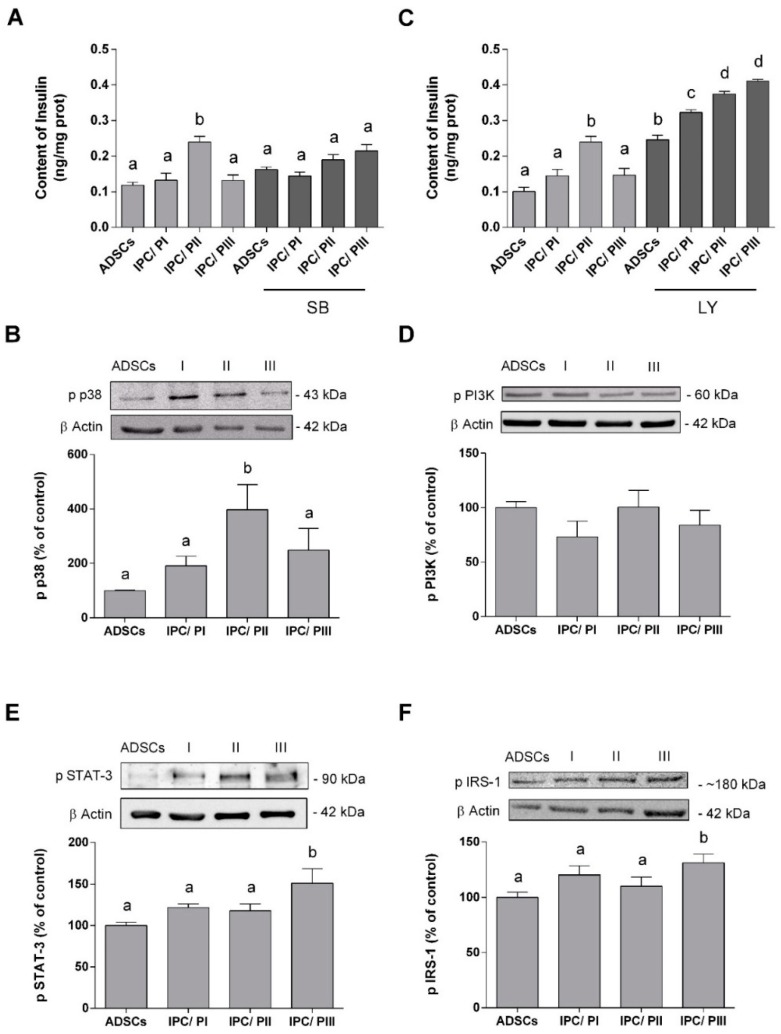
p38/MAPK, PI3K, Jak/STAT-3, and IRS-1 signaling pathways during the differentiation of ADSCs into IPCs. (**A**) Insulin content in ADSCs and IPCs (differentiated by three different protocols), incubated or not in the presence of SB203580 (at 10 μM), an inhibitor of p38/MAPK. (**B**) p38/MAPK phosphorylation in ADSCs and IPCs, as measured by Western blotting. Representative Immunoblots are shown in the inset. (**C**) Insulin content in ADSCs and IPCs (differentiated by three different protocols), incubated or not in the presence of LY294002 (at 10 μM)—a PI3K inhibitor. (**D**) PI3K phosphorylation in ADSCs and IPCs, as measured by Western blotting. Representative Immunoblots are shown in the inset. (**E**) STAT-3 phosphorylation content was analyzed by Western blotting in ADSCs and IPCs (differentiated by three different protocols). (**F**) IRS-1 phosphorylation content was analyzed by Western blotting. Data are expressed as means ± SE of 6 independent experiments performed in triplicate. Letters indicate different statistical groups by ANOVA followed by Tukey’s test, assuming *p* < 0.05.

Many signal pathways are involved in insulin expression and we investigated three of these in our IPCs: p38/MAPK [31], PI3K [32], and STAT-3 [33]. The enzyme inhibitors SB203580 and LY294002 were used to investigate the relationship between the MAPK/p38 and PI3K pathways in the differentiation process, throughout the induction period [34]. The presence of the SB203580 inhibitor prevented the increase in insulin in IPCs submitted to PII, suggesting that the p38/MAPK pathway is positively involved in cell differentiation (Figure 5A) *(p* < 0.0001; f_(7.54)_ = 6.240). Furthermore, Western blotting demonstrated an increase in the phosphorylation of p38/MAPK in these IPCs (Figure 5B) *(p* < 0.0001; f_(7.57)_ = 67.12) and no changes were observed in the content of this protein in any of the cells (data not shown).

On the other hand, incubation with the LY294002 inhibitor caused an increase in insulin expression in all IPCs, as well as in the ADSCs (Figure 5C). This effect was more pronounced in IPCs submitted to PII or PIII *(p* < 0.0001; f_(7,54)_ = 67.12). Moreover, no significant differences in PI3K phosphorylation were observed among the cells (Figure 5D) (*p* = 0.4887; f_(3,16)_ = 0.8459). 

Phosphorylation of STAT3 and the insulin-signaling pathway was assessed by Western Blotting (Figure 5E). An increase in STAT-3 phosphorylation *(p* = 0.0152; f_(3.20)_ = 4.434) occurred only in the IPCs submitted to PIII. As such, we determined whether autocrine insulin signaling was also affected by this resveratrol-induced increase in STAT-3 phosphorylation. IRS-1 phosphorylation (at serine-307) in IPCs submitted to PIII was found to be increased (Figure 5F, *p* = 0.0423; f_(3.20)_ = 3.278), but no changes were observed in the IRS-1 in any of the cells (data not shown).

### 2.6. Implant of IPCs from ADSCs in Diabetic Rats

**Figure 6:**
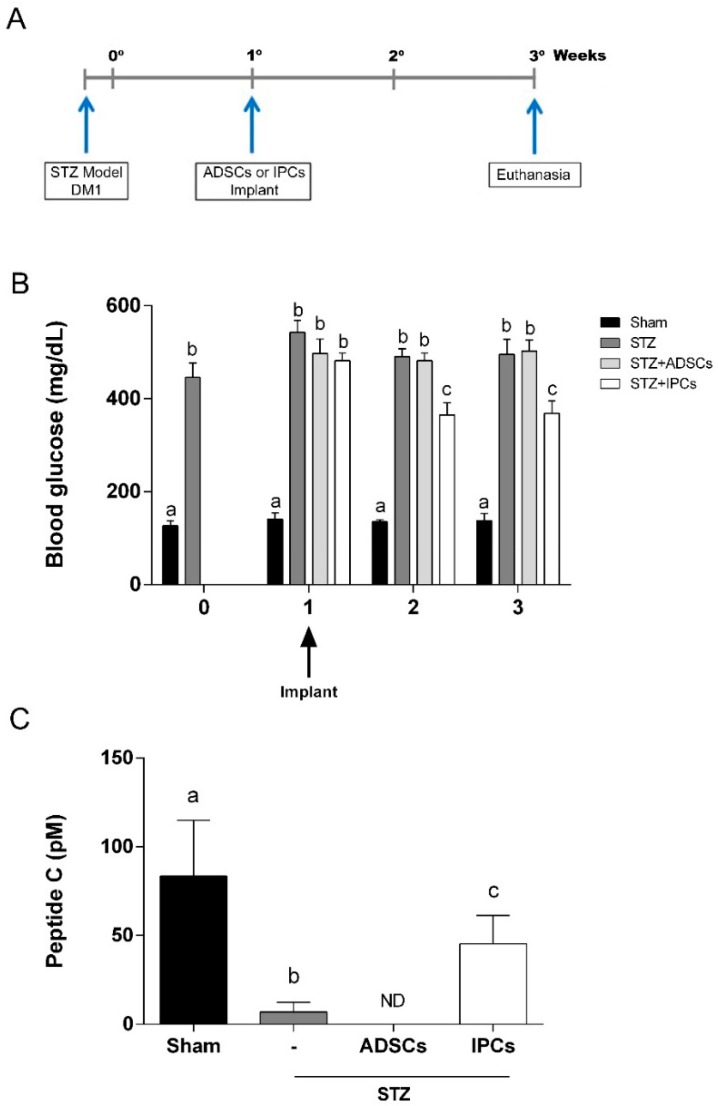
Implant of IPCs from ADSCs in diabetic rats. IPCs or ADSCs were implanted into renal subcapsular space of STZ-induced DM1 male Kyoto-Wistar rats. (**A**) Timeline of in vivo experiment, showing STZ-induced DM model and cell implant. (**B**) Blood glucose measurements before and after ADSCs and IPCs implant. (**C**) C-peptide serum content, measured by ELISA, at 2 weeks after cell implant. Data are expressed as means ± SE (*n* = 8). Letters indicate different statistical groups by ANOVA followed by Tukey’s test, assuming *p* < 0.05.

Implant of IPCs generated through protocol II (supplemented with Exendin-4 and activin A) was chose based on cells insulin production and in vitro response to glucose. IPCs cells were then implanted in the subcapsular renal region of Kyoto rats, one week after STZ-induced DM1 model (Figure 6A). In order to evaluate the effectiveness of IPCs implant, we measure the blood glucose one day, one week and two weeks afterwards. We observed that in diabetic rats that received IPCs, the hyperglycemia was reduced significantly at one and two weeks after implant (Figure 6B) (*p =* 0.0002; f_(9,112)_ = 3.928). This result suggests that IPCs do secrete insulin in vivo. Moreover, as expected, in animals transplanted with undifferentiated ADSC cells, the hyperglycemia remained unaltered.

Additionally, to confirm the in vivo insulin secretion, we evaluate the serum C-peptide in all experimental groups two weeks after the implants. Elevated levels of C-peptide were observed in IPC-treated diabetic animals when compared to untreated diabetic or ADSC-treated diabetic animals (Figure 6C) (*p* = 0.0123; f_(3, 20)_ = 4.682). However, C-peptide levels in IPC-treated diabetic rats did not reach serum levels found in sham group. 

## 3. Discussion

In recent years, MSCs derived from different tissues, including adipose tissue, have attracted attention for its use in cell regeneration research, mainly because of the abundance of available tissue and the ease of production as well as the trophic capacity of the cells. All of the characteristics of ADSCs provide the potential for these cells to differentiate into IPCs and make them a good alternative to pancreatic islet transplantation, which is considered a promising treatment for diabetes mellitus [9,13,35,36]. 

The ADSCs in this study presented functional criteria that are compatible with the identification of genuine mesenchymal stromal cells according to the International Society of Cell Therapy criteria [37]. In addition, as the mesenchymal stromal cells were isolated from the epididymal adipose tissue, the content of S100B, a calcium-binding protein characteristically expressed in this tissue [38,39] was investigated. In fact, S100B is highly expressed (and secreted) by adipose tissue [40], but was clearly not expressed in ADSCs.

There are several available protocols that can be used to differentiate ADSCs into IPCs and, based on a combination of those, we chose three protocols, considering simplicity (number of inductors), short duration and those that are currently in use, to provide a more direct comparative analysis. Our previous studies with EX-4 and resveratrol influenced this choice [41,42]. It is also important to emphasize that we look for functional insulin-secreting cells in this work rather than a pancreatic β cell phenotype, which takes in account, in addition to insulin secretion, other features such as morphology or islet aggregation (for a review, see [43]). We observed that protocols II (supplemented with EX-4 and activin A) and III (supplemented with resveratrol) generated cells with higher affinity for DTZ stain (indirect labeling of insulin presence) and pro-insulin (evaluated by Western blotting). However, a much higher content of insulin (as measured by ELISA and immunofluorescence) was observed in cells differentiated by protocol II than with the other two protocols.

The GLUT-2 transporter is involved in the detection of glucose by pancreatic β cells and in the mechanism of insulin secretion [44,45]. The production of insulin and the expression of GLUT-2 by cells confirm their differentiation and functionality as IPCs [46]. When the expression of GLUT-2 was evaluated, both protocols II and III induced increased GLUT-2 expression, although glucose uptake was increased in the IPCs that were differentiated with all three protocols, when compared with the undifferentiated cells. Moreover, the IPCs responded to glucose stimulus, but insulin secretion was higher in the IPCs differentiated by protocols II and III. Glucose withdrawal caused a decrease in insulin secretion only in the IPCs that were differentiated by protocol II, indicating that those cells were more similar to β-cells. These data reinforce the trophic role of EX-4 in the differentiation of IPCs and insulin secretion in β-cells [47]. Due to intrinsically low levels of antioxidant enzyme expression and activity, insulin-producing pancreatic β-cells are particularly susceptible to free radical attack [48], motivating us to use resveratrol due to its antioxidant properties in addition to its ability to promote cell differentiation. However, while IPC differentiation was observed, physiological glucose responsiveness was not observed in the cells that were differentiated with resveratrol.

Specific signaling pathways are involved in the initial stages IPCs differentiation induced by different protocols. Co-incubation with the SB203580 inhibitor during differentiation prevented the increase in insulin in IPCs submitted to protocol II, suggesting that the p38/MAPK pathway positively regulates the cell differentiation induced by EXE-4 and activin A. Reinforcing this idea, we observed an increase in the phosphorylation of p38/MAPK in these IPCs without any change in content. A previous study has shown that MAPKs play crucial roles in chondrogenesis and osteogenesis in MSCs [47] and are important for the proliferation and differentiation of mesenchymal stromal cells derived from dental pulp [49]. More recently, a positive role for the p38 pathway was demonstrated in the differentiation of osteogenic lineage from dental pulp [50]. EX-4 is a GLP-1 agonist and binds to the GLP-1 receptor by activating the p38/MAPK via the PKA pathway [51,52], consistent with our data regarding the differentiation of IPCs from ADSCs using EX-4. Moreover, activin A (also used in protocol II), which stimulates the proliferation of adipocyte progenitors cells but inhibits adipocyte differentiation [53], may contribute to IPC differentiation via the activation of p38/MAPK [54]. However, this hypothesis deserves further investigation.

Another signaling pathway investigated was the PI3K/Akt, where its inhibitor LY294002 increased insulin expression in all protocols for IPC differentiation; furthermore, PI3K phosphorylation did not differ among the cells. The role of the PI3K/Akt pathway in IPCs differentiation has been previously described (e.g., [55]), but some conflicting evidence exists in the literature. For example, PI3K activation was described in the differentiation of pancreatic endocrine cells [56], but an opposite effect has also been described [57]. In our study, the presence of LY94002 increased insulin expression in ADSCs, suggesting an inductive behavior of this compound per se. Moreover, the increase in the presence of this inhibitor was more pronounced in the protocols with EX-4/activin A or resveratrol. The molecular basis of this mechanism is unclear, but clearly, PI3K/Akt has a negative role in the modulation of IPC differentiation from ADSCs.

**Figure 7:**
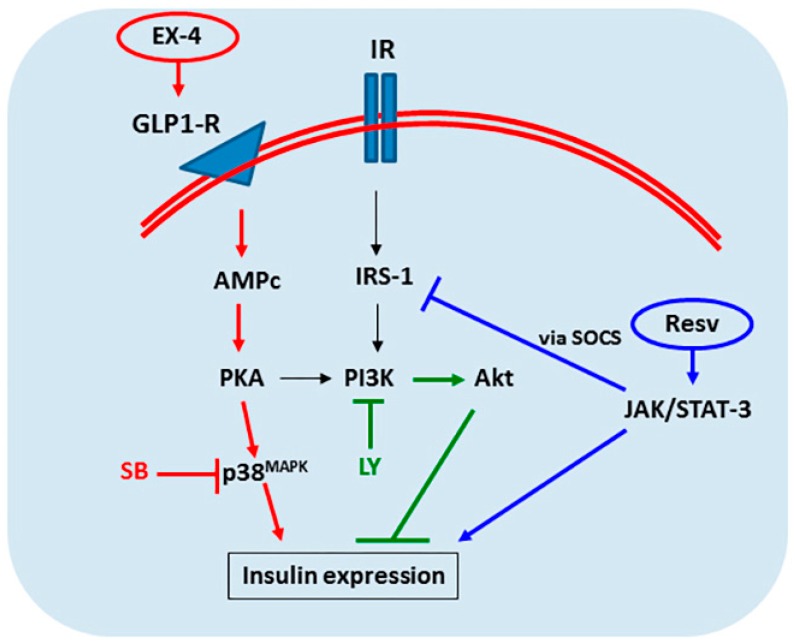
Schematic representation of some of the signaling pathways involved in the differentiation of ADSCs into IPCs. The increase in insulin expression by ADSCs is dependent on a set of signaling pathways. The MAPK/p38 (red) and Jak/STAT-3 (blue) pathways act positively on the differentiation of cells into insulin-producing cells, whereas the PI3K (green labelled) pathway acts negatively. Exendin-4 has been shown to be a good inducer of cell differentiation by enhancing p38 phosphorylation, which is inhibited by SB203580 (SB). PI3K inhibition by LY294002 (LY) augmented insulin production in all protocols of differentiation. Resveratrol (Resv) activates the Jak/STAT-3 pathway, culminating in insulin expression, but to a lesser extent when compared to exendin-4.

In contrast to the EX-4/Activin A protocol, resveratrol did not activate the p38/MAPK pathway, but activated the JAK/Stat pathway, as demonstrated by augmented STAT3 phosphorylation. This pathway has previously been reported as involved in IPC differentiation (e.g., [33]). Notably, cytokine signaling, thought to stimulate IPC differentiation, is able to activate both the p38/MAPK and Jak/STAT3 pathways. Assuming that STAT3 activation could affect autocrine insulin signaling via SOCS [58,59], we investigated IRS-1 phosphorylation in IPCs during differentiation. In fact, in protocol III, which employed resveratrol, IRS-1 phosphorylation at serine-307 was increased, suggesting a lower sensitivity to insulin and insulin-like activators. Resveratrol is apparently able to differentiate MSCs into adipocytes and to work as an insulin agonist in these cells [60]. Thus, our results suggest that resveratrol can also differentiate ADSCs into IPCs, but that these IPCs exhibit lower autocrine sensitivity to insulin (and other insulin-like activators) as well as lower responsiveness to glucose withdrawal. Figure 7 summarizes the signaling pathways studied herein; however, the involvement of other signaling pathways cannot be ruled out. 

Based on in vitro insulin production and glucose responsivity we decided to implant IPCs differentiated by protocol II. Implanted IPCs in the renal subcapsular region were able to secrete functional insulin, confirmed through the presence of serum C-peptide and the reduction of hyperglycemia. The renal subcapsular space is a site commonly used in experimental cell implantation [8,15,61], being a potential approach for clinical application. Our findings indicate that adipose-derived IPCs, obtained with a simple and fast protocol was able to reduce (although partially) the hyperglycemia in DM1. According to the literature, other works also had obtained the similar effect on glycemia control, even using longer protocols of differentiation and adding more inductors (e.g., [62]).

Some limitations of our study deserve to be commented. Firstly, we are aware that the time of cell differentiation (three days for protocols II and III versus seven days for protocol I), as well as the presence of FBS (protocol I) complicate analysis and comparisons between the different media of differentiation. Nevertheless, the medium with EX-4 and activin A has consistently been found to generate IPCs. Secondly, no differentiation protocol results in fully differentiated cells [15,63]. Therefore, our implant strategy (4 × 10^6^ differentiated cells/rat) may not had had the enough IPCs for a complete reversion in hyperglycemia, although it had contributed to a significantly reduction. Perhaps larger implants or implants more enriched with differentiated cells would improve the results in further investigations. Thirdly, despite of the promising results, we evaluated the efficacy of the implants only one and two weeks after surgery. Future investigations will determine whether these cells are able to control glycemia for long-term periods or at least to diminish some of the global consequences of diabetes mellitus, such as the neurodegenerative alterations and cognitive deficits observed in patients and experimental models. 

## 4. Material and Methods

### 4.1. Animals

ADSCs were isolated from abdominal adipose (8-week-old) obtained from male Kyoto-Wistar rats maintained at our breeding colony (Institute of Cardiology of Rio Grande do Sul, Experimental Center, Porto Alegre, Brazil). The animals were maintained under controlled light and environmental conditions (12 h light/12 h dark cycle at a constant temperature of 22 ± 1 °C) with free access to commercial chow and water. All animal experiments were carried out in accordance with the National Institutes of Health Guide for the Care and Use of Laboratory Animals and were approved by the Federal University of Rio Grande do Sul Animal Care and Use Committee (process number 30626, 17 August 2016).

### 4.2. Chemicals

Fetal bovine serum (FBS), Dulbecco’s modified Eagle medium (DMEM), and other materials for cell culture were purchased from Gibco BRL (Carlbad, CA, USA). D-[3-^3^H] deoxy-glucose (20 Ci/mmol) was purchased from Perkin-Elmer (Boston, MA, USA). Cytochalasin B (Cyt B), 4-(2-hydroxyethyl)-piperazine-1-ethanesulfonic acid (HEPES), S100B protein, anti-S100B antibody (SH-B1), o-phenylenediamine (OPD), Anti-S100B antibody (clone SH-B) and antibodies for blotting: anti-p PI3K was purchased from Cell Signaling (Frankfurt, Germany); anti-p p38 and anti-GLUT2 were from Sigma-Aldrich (St. Louis, MO, USA); anti-insulin and anti-p STAT3 were purchased from Santa Cruz Biotechnology (Santa Cruz, CA, USA); and anti-β-actin and anti-p IRS were from EMD Millipore (Darmstadt, Germany). Other reagents were purchased from local commercial suppliers (Sulquímica, Labsul or Biogen; Porto Alegre, Brazil).

### 4.3. Isolation and Expansion of Rat Adipose Tissue-Derived Stromal Cells (ADSCs)

Epididymal fat was collected aseptically from the animals and minced into small pieces. The fragments were digested with 1.5 mg/mL of collagenase type I (Sigma) and diluted in DMEM without serum for 20 min at 37 °C, and then added DMEM 10% FBS with 0.1 mg/mL streptomycin/100 U/mL penicillin for interruption of enzymatic activity. After centrifugation at 1500 rpm for 5 min, each pellet was resuspended using DMEN 10% FBS in a wet chamber at 37 °C/5% CO_2_ until cell confluence. The cells were detached using 0.05% trypsin-EDTA solution. In all experiments, were used cells at the fourth passage [64]. 

### 4.4. Characterization of ADSCs

Surface marker analysis of the isolated MSCs was performed by incubation with phycoerythrin-conjugated antibodies against murine CD29, CD44, CD90, CD45, CD31, and MHC II for 30 min at 4 °C. The cells were analyzed using a FACSAria III cytometer (Becton Dickinson, San Jose, CA, USA) equipped with a 488nm argon laser, and graphs were generated in WinMDI 9.2 software. The adipogenic and osteogenic differentiation of the MSCs was performed according to previously published protocols [65]. After 4% paraformaldehyde fixation, the calcium deposition and lipid droplets were stained with Alizarin Red S and Oil Red O solution, respectively. 

### 4.5. ELISA for S100B

S100B contents were determined by ELISA according previously work [66]. In short, 50 μL of sample plus 50 μL of Tris buffer were incubated on a microtiter plate previously coated with anti-S100B monoclonal antibody (SH-B1, from Sigma) for 2 h. Anti-S100 polyclonal antibody (from DAKO) was incubated for 30 min and then peroxidase-conjugated anti-rabbit antibody was added for a further 30 min. The color reaction with o-phenylenediamine was measured at 492 nm. The standard S100B curve ranged from 0.002 to 1 ng/mL.

### 4.6. Protocol for Differentiation of Mesenchymal Stromal Cells into Insulin-Producing Cells (IPCs)

In each of the protocols tested, including the control (DMEM serum free), the cells used were in passage 4 (P4), cultured in triplicate in a 6-well plate (TPP), and with a confluence of greater than 80% (~4 × 10^5^ cells/mL). Cells were incubated in a humidified atmosphere at 37 °C and 5% CO_2_. Protocol I: Insulin-Producing Cells (IPC/PI) (adapted from Chen et al., 2004 [5]). Cells were maintained in DMEM-F12 (Dulbecco’s Modified Eagle Medium: Nutrient Mixture F-12, Gibco) for 7 days with 1% penicillin/streptomycin, 2% FBS supplemented with 10 nM nicotinamide. Protocol II: Insulin-Producing Cells (IPC/PII) (adapted from Timper et al., 2006 [67]). Cells were maintained for 3 days in serum-free DMEM-F12 with 1% penicillin/streptomycin, supplemented with 10 nM nicotinamide (Sigma), 10 ng/mL activin-A (Sigma), and 10 nM exendin-4 (Sigma, St. Louis, MO, USA). Protocol III: Insulin-Producing Cells (IPC/PIII). Cells were maintained for 3 days, in serum-free DMEM-F12 with 1% penicillin/streptomycin, supplemented with 10 nM nicotinamide (Sigma) and 25 μM resveratrol [60]. For the evaluation of the different signaling pathways that are involved in cell differentiation, the protocols listed above were incubated (serum-free DMEM) concomitantly with the following specific inhibitors: 10 μM LY294002 (PI3K inhibitor) or 10 μM SB203580 (p38 inhibitor) [68].

### 4.7. Staining with Dithizone

Dithizone (DTZ) is a zinc ion-chelating agent (Zn^2+^) that is present in the insulin granules, and selectively stains them. In order to identify the insulin-producing cells, 10 μg/mL of sterile DTZ from a stock solution was added to the culture medium, as described by Shiroi et al., 2002 [69].

### 4.8. Immunofluorescence

The cells were cultured on circular glass cover slips. After treatment (and control) protocols, cells were fixed for 20 min with 4% paraformaldehyde in phosphate buffer (PBS), washed with PBS and permeabilized for 20 min in PBS containing 0.2% Triton X-100. The cells were then blocked for 1 h with PBS containing 5% bovine serum albumin and incubated overnight with anti-insulin monoclonal antibody (clone H-86) at a 1:200 dilution, or monoclonal anti-GLUT2 at a 1:500 dilution. Following overnight incubation, cells were washed in PBS/triton 0.2% (3 × 5 min) and incubated for 2 h with the respective secondary antibody at a 1:1000 dilution—Alexa Fluor 528 (goat anti-mouse-IgG; red fluorescence) and Alexa 488 (goat anti-rabbit-IgG; green fluorescence). After washing, cells were incubated with 4′,6-diamidino-2-phenylindole (DAPI, Sigma) for 10 min. Images were captured using an Olympus BX51 phase-contrast fluorescent microscope (Olympus, Japan) and transferred to a computer with a digital camera and Fluoviewer 3.1 FV1000 software for analysis [70].

### 4.9. Insulin Content

Measurement of insulin content was performed using commercial Rat/Mouse ELISAs (Millipore), according to the manufacturer’s instructions.

### 4.10. Evaluation of Glucose-Stimulated Insulin Secretion

Insulin secretion into the cell culture supernatant was evaluated using the commercial Rat Insulin ELISA (Millipore), according to the manufacturer’s instructions. Briefly, the IPCs were incubated for 1 hour, three times. Each incubation step was performed with the collection of the medium at the end for the evaluation of insulin secretion. Cells were washed carefully three times with DPBS and then incubated with DMEM without glucose—DMEM F12 (17.5 mM glucose)—and DMEM without glucose, respectively. The three steps each lasted one hour and cell supernatants were collected every time for evaluation.

### 4.11. Glucose Uptake Assay

Glucose uptake was performed as previously described [34,70], with some modifications. The cells were incubated with DMEM without glucose during 1 hour at 37 °C. Then, the medium was removed and cells were incubated at 35 °C in a Hank’s balanced salt solution (HBSS). The assay started by the addition of 0.1 μCi/well D-[2,3-^3^H] deoxy-glucose during 15 min. The incubation was stopped by removing the medium and rinsing the slices twice with ice-cold HBSS. The slices were then lysed in a 0.5 M NaOH solution. Glucose uptake was calculated by subtracting the nonspecific uptake, obtained using the glucose transporter inhibitor, cytochalasin B (25 μM), from the total uptake. Radioactivity was measured using a scintillation counter. Results are expressed as nmol/mg protein/min.

### 4.12. Western Blot Analysis 

Samples were prepared in lysis buffer (containing 62.5 mM Tris-HCl, pH 6.8, 10% (*v*/*v*), 2% (*w*/*v*) SDS, glycerol, 5% (*w*/*v*) β-mercaptoethanol, and 0.002% bromphenol blue), and analyzed by SDS-PAGE on 12% (*w*/*v*) acrylamide gel before electro transferring onto nitrocellulose membranes. Membranes were incubated in TBS-T (20 mmol/L Tris-HCl, pH 7.5, 137 mmol/L NaCl, 0.05% (*v*/*v*) Tween 20) containing 5% (*w*/*v*) bovine serum albumin (BSA) for 1 h at room temperature. After, the membranes were incubated overnight with the respective primary antibodies—anti-insulin, anti-GLUT-2, anti-p IRS-1, anti-p STAT3, anti-p P38, anti-p PI3K (dilution 1:1000), and anti-β-actin (dilution 1:5000)—rinsed with TBS-T, and exposed to horseradish peroxidase-linked anti-IgG antibodies for 2 h at room temperature. Detection of chemiluminescent bands were made using Image Quant LAS4000 GE Healthcare, and densitometric analyses were performed using Image-J software. Results are expressed as percentages of the control. 

### 4.13. DM1 Model and Surgical Procedure–IPCs Implant

DM 1 model was induced by intraperitoneal injection of streptozotocin (STZ) (60 mg/kg in citrate buffer, pH 4.5). Animals with a glycemic greater than 250 mg/dL will be considered diabetic. A schematic representation of the experimental protocol is shown in Figure 6A.

Adult male Kyoto-Wistar rats were divided in four groups: Group I (Sham group): included 8 rats injected with vehicle via intraperitoneal (IP) injection and passed through all surgical steps except transplantation. Group II, III, and IV: each group included 8 rats injected once with streptozotocin (60 mg/kg body weight) via IP injection; Group II (diabetic group—STZ): passed through all surgical steps except transplantation. Group III (STZ + ADSCs group): ADSCs were transplanted into subcapsular renal (4 × 10^6^ cells/ rat), after 1-week DM 1 induction. Group IV (STZ + IPCs group): IPCs were transplanted into subcapsular renal (4 × 10^6^ cells/ rat), after 1-week DM 1 induction.

Surgical procedure for subcapsular renal transplantation. Animals were anesthetized with ketamine/xylazine (75 and 10 mg/kg, respectively, IP. After trichotomy in the left inferior dorsolateral region, the kidney was exposed through a 1.5 cm incision and, with the aid of a straight thin forceps, 4 × 10^6^ cells were implanted under the renal capsule. Sutured the two planes with suture wire 7.0, the animal was placed on heating plate and monitored until it presents reactions to external stimuli [71].

### 4.14. Protein Determination

Protein content was measured by Lowry’s method with some modifications, using bovine serum albumin as the standard [72].

### 4.15. Statistical Analysis

All in vitro experiments were performed in triplicate in at least six independent experiments and the data represent means ± SD. Statistical comparisons between different groups were tested by one-way ANOVA followed by the Tukey’s test. For the S100B content, data are presented as means ± S.E.M. and statistically evaluated by Student’s *t*-test. For the evaluation of glucose-stimulated insulin secretion, statistical comparisons between different groups were made using repeated measures ANOVA followed by Tukey’s test. All in vivo experiments were expressed as means ± SE (*n* = 8) and were tested by one-way ANOVA followed by Tukey’s test. Values of *p* < 0.05 were considered significant. All analyses were performed using the Graphpad Prism software version 6 (La Jolla, CA, USA). 

## 5. Conclusions

In summary, our data showed that is obtaining IPCs from ADSCs is viable and relatively easy using a short-duration protocol. Protocols using EX-4 and activin A or resveratrol were able to generate IPCs more efficiently, as shown by their expression of biomarkers (e.g., insulin and GLUT-2), but only the protocol employing EX-4 and activin A was able to yield IPCs exhibiting a response to glucose that was more similar to that of β-cells. Moreover, p38/MAPK is involved in the process of IPC differentiation and possibly in the responsiveness to glucose. Resveratrol also promoted IPC differentiation independently of p38/MAPK, possibly by Jak/STAT3 activation, but these IPCs cells did not respond to glucose withdrawal and exhibited lower sensitivity to the insulin-substrate receptor 1, an adapter protein that binds insulin and insulin-like receptors. Our data contribute to the understanding of the generation of IPCs from autologous adipose tissue and preparation of effective implants of these cells, reinforcing the use of ADSCs in regenerative medicine for DM1. 

## Data Availability

The data used to support the findings of this study are available from the corresponding author upon request.

## References

[1] Van Belle T.L., Coppieters K.T., von Herrath M.G. (2011). Type 1 diabetes: Etiology, immunology, and therapeutic strategies. Physiol. Rev..

[2] Chhabra P., Brayman K.L. (2013). Stem cell therapy to cure type 1 diabetes: From hype to hope. Stem Cells Transl. Med..

[3] Aguayo-Mazzucato C., Bonner-Weir S. (2018). Pancreatic β Cell Regeneration as a Possible Therapy for Diabetes. Cell Metab..

[4] Daley G.Q. (2012). The promise and perils of stem cell therapeutics. Cell Stem Cell.

[5] Chen L.-B., Jiang X.-B., Yang L. (2004). Differentiation of rat marrow mesenchymal stem cells into pancreatic islet beta-cells. World J. Gastroenterol..

[6] Karnieli O., Izhar-Prato Y., Bulvik S., Efrat S. (2007). Generation of insulin-producing cells from human bone marrow mesenchymal stem cells by genetic manipulation. Stem Cells Dayt. Ohio.

[7] Oh S.-H., Muzzonigro T.M., Bae S.-H., LaPlante J.M., Hatch H.M., Petersen B.E. (2004). Adult bone marrow-derived cells trans-differentiating into insulin-producing cells for the treatment of type I diabetes. Lab. Investig. J. Tech. Methods Pathol..

[8] Paz A.H., Salton G.D., Ayala-Lugo A., Gomes C., Terraciano P., Scalco R., Laurino C.C.F.C., Passos E.P., Schneider M.R., Meurer L. (2011). Betacellulin overexpression in mesenchymal stem cells induces insulin secretion in vitro and ameliorates streptozotocin-induced hyperglycemia in rats. Stem Cells Dev..

[9] Chandra V., Swetha G., Muthyala S., Jaiswal A.K., Bellare J.R., Nair P.D., Bhonde R.R. (2011). Islet-like cell aggregates generated from human adipose tissue derived stem cells ameliorate experimental diabetes in mice. PLoS ONE.

[10] Francese R., Fiorina P. (2010). Immunological and regenerative properties of cord blood stem cells. Clin. Immunol. Orlando Fla.

[11] Domínguez-Bendala J., Lanzoni G., Inverardi L., Ricordi C. (2012). Concise Review: Mesenchymal Stem Cells for Diabetes. STEM CELLS Transl. Med..

[12] Kim B., Yoon B.S., Moon J.H., Kim J., Jun E.K., Lee J.H., Kim J.S., Baik C.S., Kim A., Whang K.Y. (2012). Differentiation of human labia minora dermis-derived fibroblasts into insulin-producing cells. Exp. Mol. Med..

[13] Khorsandi L., Saremy S., Khodadadi A., Dehbashi F. (2016). Effects of Exendine-4 on The Differentiation of Insulin Producing Cells from Rat Adipose-Derived Mesenchymal Stem Cells. Cell J..

[14] Nejad-Dehbashi F., Hashemitabar M., Orazizadeh M., Bahramzadeh S., Shahhosseini Pourshoushtary E., Khorsandi L. (2014). The effects of exendine-4 on insulin producing cell differentiation from rat bone marrow-derived mesenchymal stem cells. Cell J..

[15] Xin Y., Jiang X., Wang Y., Su X., Sun M., Zhang L., Tan Y., Wintergerst K.A., Li Y., Li Y. (2016). Insulin-Producing Cells Differentiated from Human Bone Marrow Mesenchymal Stem Cells In Vitro Ameliorate Streptozotocin-Induced Diabetic Hyperglycemia. PLoS ONE.

[16] Xu G., Stoffers D.A., Habener J.F., Bonner-Weir S. (1999). Exendin-4 stimulates both beta-cell replication and neogenesis, resulting in increased beta-cell mass and improved glucose tolerance in diabetic rats. Diabetes.

[17] Greig N.H., Holloway H.W., De Ore K.A., Jani D., Wang Y., Zhou J., Garant M.J., Egan J.M. (1999). Once daily injection of exendin-4 to diabetic mice achieves long-term beneficial effects on blood glucose concentrations. Diabetologia.

[18] Nardin P., Zanotto C., Hansen F., Batassini C., Gasparin M.S., Sesterheim P., Gonçalves C.-A. (2016). Peripheral Levels of AGEs and Astrocyte Alterations in the Hippocampus of STZ-Diabetic Rats. Neurochem. Res..

[19] Das S., Das D.K. (2007). Anti-inflammatory responses of resveratrol. Inflamm. Allergy Drug Targets.

[20] Kao C.-L., Tai L.-K., Chiou S.-H., Chen Y.-J., Lee K.-H., Chou S.-J., Chang Y.-L., Chang C.-M., Chen S.-J., Ku H.-H. (2010). Resveratrol promotes osteogenic differentiation and protects against dexamethasone damage in murine induced pluripotent stem cells. Stem Cells Dev..

[21] Liu H., Zhang S., Zhao L., Zhang Y., Li Q., Chai X., Zhang Y. (2016). Resveratrol Enhances Cardiomyocyte Differentiation of Human Induced Pluripotent Stem Cells through Inhibiting Canonical WNT Signal Pathway and Enhancing Serum Response Factor-miR-1 Axis. Stem Cells Int..

[22] Dai Z., Li Y., Quarles L.D., Song T., Pan W., Zhou H., Xiao Z. (2007). Resveratrol enhances proliferation and osteoblastic differentiation in human mesenchymal stem cells via ER-dependent ERK1/2 activation. Phytomedicine Int. J. Phytother. Phytopharm..

[23] Ding H., Xu X., Qin X., Yang C., Feng Q. (2016). Resveratrol promotes differentiation of mouse embryonic stem cells to cardiomyocytes. Cardiovasc. Ther..

[24] Baeyens L., Bouwens L. (2008). Can beta-cells be derived from exocrine pancreas?. Diabetes Obes. Metab..

[25] Koblas T., Leontovyč I., Zacharovová K., Berková Z., Kříž J., Girman P., Saudek F. (2012). Activation of the Jak/Stat signalling pathway by leukaemia inhibitory factor stimulates trans-differentiation of human non-endocrine pancreatic cells into insulin-producing cells. Folia Biol..

[26] Valdez I.A., Dirice E., Gupta M.K., Shirakawa J., Teo A.K.K., Kulkarni R.N. (2016). Proinflammatory Cytokines Induce Endocrine Differentiation in Pancreatic Ductal Cells via STAT3-Dependent NGN3 Activation. Cell Rep..

[27] Lemper M., Leuckx G., Heremans Y., German M.S., Heimberg H., Bouwens L., Baeyens L. (2015). Reprogramming of human pancreatic exocrine cells to β-like cells. Cell Death Differ..

[28] Makeeva N., Roomans G.M., Welsh N. (2006). Role of TAB1 in nitric oxide-induced p38 activation in insulin-producing cells. Int. J. Biol. Sci..

[29] Watanabe H., Saito H., Nishimura H., Ueda J., Evers B.M. (2008). Activation of phosphatidylinositol-3 kinase regulates pancreatic duodenal homeobox-1 in duct cells during pancreatic regeneration. Pancreas.

[30] Mokhtari D., Al-Amin A., Turpaev K., Li T., Idevall-Hagren O., Li J., Wuttke A., Fred R.G., Ravassard P., Scharfmann R. (2013). Imatinib mesilate-induced phosphatidylinositol 3-kinase signalling and improved survival in insulin-producing cells: Role of Src homology 2-containing inositol 5’-phosphatase interaction with c-Abl. Diabetologia.

[31] Kemp D.M., Habener J.F. (2001). Insulinotropic hormone glucagon-like peptide 1 (GLP-1) activation of insulin gene promoter inhibited by p38 mitogen-activated protein kinase. Endocrinology.

[32] Lee H.-J., Choi Y.-J., Park S.-Y., Kim J.-Y., Won K.-C., Son J.-K., Kim Y.-W. (2015). Hexane Extract of Orthosiphon stamineus Induces Insulin Expression and Prevents Glucotoxicity in INS-1 Cells. Diabetes Metab. J..

[33] Velayos T., Martínez R., Alonso M., Garcia-Etxebarria K., Aguayo A., Camarero C., Urrutia I., Martínez de LaPiscina I., Barrio R., Santin I. (2017). An Activating Mutation in STAT3 Results in Neonatal Diabetes Through Reduced Insulin Synthesis. Diabetes.

[34] Wartchow K.M., Tramontina A.C., de Souza D.F., Biasibetti R., Bobermin L.D., Gonçalves C.-A. (2016). Insulin Stimulates S100B Secretion and These Proteins Antagonistically Modulate Brain Glucose Metabolism. Neurochem. Res..

[35] Chen L., Tredget E.E., Wu P.Y.G., Wu Y. (2008). Paracrine factors of mesenchymal stem cells recruit macrophages and endothelial lineage cells and enhance wound healing. PLoS ONE.

[36] Gao X., Song L., Shen K., Wang H., Qian M., Niu W., Qin X. (2014). Bone marrow mesenchymal stem cells promote the repair of islets from diabetic mice through paracrine actions. Mol. Cell. Endocrinol..

[37] Bourin P., Bunnell B.A., Casteilla L., Dominici M., Katz A.J., March K.L., Redl H., Rubin J.P., Yoshimura K., Gimble J.M. (2013). Stromal cells from the adipose tissue-derived stromal vascular fraction and culture expanded adipose tissue-derived stromal/stem cells: A joint statement of the International Federation for Adipose Therapeutics and Science (IFATS) and the International Society for Cellular Therapy (ISCT). Cytotherapy.

[38] Hidaka H., Endo T., Kawamoto S., Yamada E., Umekawa H., Tanabe K., Hara K. (1983). Purification and characterization of adipose tissue S-100b protein. J. Biol. Chem..

[39] Michetti F., Dell’Anna E., Tiberio G., Cocchia D. (1983). Immunochemical and immunocytochemical study of S-100 protein in rat adipocytes. Brain Res..

[40] Gonçalves C.A., Leite M.C., Guerra M.C. (2010). Adipocytes as an Important Source of Serum S100B and Possible Roles of This Protein in Adipose Tissue. Cardiovasc. Psychiatry Neurol..

[41] Bellaver B., Bobermin L.D., Souza D.G., Rodrigues M.D.N., de Assis A.M., Wajner M., Gonçalves C.-A., Souza D.O., Quincozes-Santos A. (2016). Signaling mechanisms underlying the glioprotective effects of resveratrol against mitochondrial dysfunction. Biochim. Biophys. Acta.

[42] Zanotto C., Simão F., Gasparin M.S., Biasibetti R., Tortorelli L.S., Nardin P., Gonçalves C.-A. (2017). Exendin-4 Reverses Biochemical and Functional Alterations in the Blood-Brain and Blood-CSF Barriers in Diabetic Rats. Mol. Neurobiol..

[43] Balboa D., Saarimäki-Vire J., Otonkoski T. (2019). Concise Review: Human Pluripotent Stem Cells for the Modeling of Pancreatic β-Cell Pathology. Stem Cells.

[44] Kellett G.L., Brot-Laroche E., Mace O.J., Leturque A. (2008). Sugar absorption in the intestine: The role of GLUT2. Annu. Rev. Nutr..

[45] Olson A.L., Pessin J.E. (1996). Structure, function, and regulation of the mammalian facilitative glucose transporter gene family. Annu. Rev. Nutr..

[46] Tang D.-Q., Cao L.-Z., Burkhardt B.R., Xia C.-Q., Litherland S.A., Atkinson M.A., Yang L.-J. (2004). In vivo and in vitro characterization of insulin-producing cells obtained from murine bone marrow. Diabetes.

[47] Li J., Zhao Z., Liu J., Huang N., Long D., Wang J., Li X., Liu Y. (2010). MEK/ERK and p38 MAPK regulate chondrogenesis of rat bone marrow mesenchymal stem cells through delicate interaction with TGF-beta1/Smads pathway. Cell Prolif..

[48] Dinić S., Grdović N., Uskoković A., Đorđević M., Mihailović M., Jovanović J.A., Poznanović G., Vidaković M. (2016). CXCL12 protects pancreatic β-cells from oxidative stress by a Nrf2-induced increase in catalase expression and activity. Proc. Jpn. Acad. Ser. B Phys. Biol. Sci..

[49] Zhang H., Liu S., Zhou Y., Tan J., Che H., Ning F., Zhang X., Xun W., Huo N., Tang L. (2012). Natural mineralized scaffolds promote the dentinogenic potential of dental pulp stem cells via the mitogen-activated protein kinase signaling pathway. Tissue Eng. Part A.

[50] Ba P., Duan X., Fu G., Lv S., Yang P., Sun Q. (2017). Differential effects of p38 and Erk1/2 on the chondrogenic and osteogenic differentiation of dental pulp stem cells. Mol. Med. Rep..

[51] Roussel M., Mathieu J., Dalle S. (2016). Molecular mechanisms redirecting the GLP-1 receptor signalling profile in pancreatic β-cells during type 2 diabetes. Horm. Mol. Biol. Clin. Investig..

[52] Yang J.-L., Chen W.-Y., Chen Y.-P., Kuo C.-Y., Chen S.-D. (2016). Activation of GLP-1 Receptor Enhances Neuronal Base Excision Repair via PI3K-AKT-Induced Expression of Apurinic/Apyrimidinic Endonuclease 1. Theranostics.

[53] Zaragosi L.-E., Wdziekonski B., Villageois P., Keophiphath M., Maumus M., Tchkonia T., Bourlier V., Mohsen-Kanson T., Ladoux A., Elabd C. (2010). Activin a plays a critical role in proliferation and differentiation of human adipose progenitors. Diabetes.

[54] Hu W., Lu H., Wang S., Yin W., Liu X., Dong L., Chiu R., Shen L., Lu W.-J., Lan F. (2016). Suppression of Nestin reveals a critical role for p38-EGFR pathway in neural progenitor cell proliferation. Oncotarget.

[55] Mao G.-H., Lu P., Wang Y.-N., Tian C.-G., Huang X.-H., Feng Z.-G., Zhang J.-L., Chang H.-Y. (2017). Role of PI3K p110β in the differentiation of human embryonic stem cells into islet-like cells. Biochem. Biophys. Res. Commun..

[56] Kohn A.D., Summers S.A., Birnbaum M.J., Roth R.A. (1996). Expression of a constitutively active Akt Ser/Thr kinase in 3T3-L1 adipocytes stimulates glucose uptake and glucose transporter 4 translocation. J. Biol. Chem..

[57] Hori Y., Rulifson I.C., Tsai B.C., Heit J.J., Cahoy J.D., Kim S.K. (2002). Growth inhibitors promote differentiation of insulin-producing tissue from embryonic stem cells. Proc. Natl. Acad. Sci. USA.

[58] Fan N., Sun H., Wang Y., Zhang L., Xia Z., Peng L., Hou Y., Shen W., Liu R., Peng Y. (2014). Midkine, a Potential Link between Obesity and Insulin Resistance. PLoS ONE.

[59] Galic S., Sachithanandan N., Kay T.W., Steinberg G.R. (2014). Suppressor of cytokine signalling (SOCS) proteins as guardians of inflammatory responses critical for regulating insulin sensitivity. Biochem. J..

[60] Caldarelli I., Speranza M.C., Bencivenga D., Tramontano A., Borgia A., Pirozzi A.V.A., Perrotta S., Oliva A., Della Ragione F., Borriello A. (2015). Resveratrol mimics insulin activity in the adipogenic commitment of human bone marrow mesenchymal stromal cells. Int. J. Biochem. Cell Biol..

[61] Gabr M.M., Zakaria M.M., Refaie A.F., Ismail A.M., Abou-El-Mahasen M.A., Ashamallah S.A., Khater S.M., El-Halawani S.M., Ibrahim R.Y., Uin G.S. (2013). Insulin-producing cells from adult human bone marrow mesenchymal stem cells control streptozotocin-induced diabetes in nude mice. Cell Transplant..

[62] Kondo Y., Toyoda T., Ito R., Funato M., Hosokawa Y., Matsui S., Sudo T., Nakamura M., Okada C., Zhuang X. (2017). Identification of a small molecule that facilitates the differentiation of human iPSCs/ESCs and mouse embryonic pancreatic explants into pancreatic endocrine cells. Diabetologia.

[63] Gabr M.M., Zakaria M.M., Refaie A.F., Khater S.M., Ashamallah S.A., Ismail A.M., El-Badri N., Ghoneim M.A. (2014). Generation of insulin-producing cells from human bone marrow-derived mesenchymal stem cells: Comparison of three differentiation protocols. BioMed Res. Int..

[64] Meirelles L.d.S., Nardi N.B. (2003). Murine marrow-derived mesenchymal stem cell: Isolation, in vitro expansion, and characterization. Br. J. Haematol..

[65] Da Silva Meirelles L., Chagastelles P.C., Nardi N.B. (2006). Mesenchymal stem cells reside in virtually all post-natal organs and tissues. J. Cell Sci..

[66] Leite M.C., Galland F., Brolese G., Guerra M.C., Bortolotto J.W., Freitas R., de Almeida L.M.V., Gottfried C., Gonçalves C.-A. (2008). A simple, sensitive and widely applicable ELISA for S100B: Methodological features of the measurement of this glial protein. J. Neurosci. Methods.

[67] Timper K., Seboek D., Eberhardt M., Linscheid P., Christ-Crain M., Keller U., Müller B., Zulewski H. (2006). Human adipose tissue-derived mesenchymal stem cells differentiate into insulin, somatostatin, and glucagon expressing cells. Biochem. Biophys. Res. Commun..

[68] De Souza D.F., Wartchow K., Hansen F., Lunardi P., Guerra M.C., Nardin P., Gonçalves C.-A. (2013). Interleukin-6-induced S100B secretion is inhibited by haloperidol and risperidone. Prog. Neuropsychopharmacol. Biol. Psychiatry.

[69] Shiroi A., Yoshikawa M., Yokota H., Fukui H., Ishizaka S., Tatsumi K., Takahashi Y. (2002). Identification of insulin-producing cells derived from embryonic stem cells by zinc-chelating dithizone. Stem Cells Dayt. Ohio.

[70] Pellerin L., Magistretti P.J. (1994). Glutamate uptake into astrocytes stimulates aerobic glycolysis: A mechanism coupling neuronal activity to glucose utilization. Proc. Natl. Acad. Sci. USA.

[71] Li D., Hao J., Yuan Y.-H., Yun S.H., Feng J.-B., Dai L.-J., Warnock G.L. (2011). Pancreatic islet transplantation to the renal subcapsule in mice. Protoc. Exch..

[72] Peterson G.L. (1977). A simplification of the protein assay method of Lowry et al. which is more generally applicable. Anal. Biochem..

